# Validation and convergent validity of the Boston cognitive assessment (BOCA) in an Italian population: a comparative study with the Montreal cognitive assessment (MoCA) in Alzheimer’s disease spectrum

**DOI:** 10.1007/s10072-024-07775-3

**Published:** 2024-09-24

**Authors:** Alessandro Padovani, Salvatore Caratozzolo, Alice Galli, Luca Crosani, Silvio Zampini, Maura Cosseddu, Rosanna Turrone, Andrea Zancanaro, Bianca Gumina, Barbara Vicini-Chilovi, Alberto Benussi, Andrey Vyshedskiy, Andrea Pilotto

**Affiliations:** 1 Neurology Unit, Department of Clinical and Experimental Sciences, University of Brescia, Brescia, Italy; 2 Department of continuity of care and frailty, Neurology Unit, ASST Spedali Civili Hospital, Brescia, Italy; 3 Neurobiorepository and Laboratory of advanced biological markers, University of Brescia and ASST Spedali Civili Hospital, Brescia, Italy; 4 Laboratory of digital Neurology and biosensors, University of Brescia, Brescia, Italy; 5 Brain Health Center, University of Brescia, Brescia, Italy; 6 Boston University, 9 Michael Rd, Boston, MA 02135 USA; 7 Alzheimer’s Light, Miami, FL USA; 8 Neurology Unit, University of Brescia, P. le Spedali Civili 1, Brescia, 25123 Italy

**Keywords:** Digital cognitive testing, Alzheimer’s disease, MoCa, BOCA, Amyloid

## Abstract

**Background:**

The Boston Cognitive Assessment (BOCA) is a self-administered online test developed for cognitive screening and longitudinal monitoring of brain health in an aging population. The study aimed to validate BOCA in an Italian population and to investigate the convergent validity with the Montreal Cognitive Assessment (MOCA) in healthy ageing population and patients within the Alzheimer Disease spectrum.

**Methods:**

BOCA was administered to 150 participants, including cognitively healthy controls (HC, *n* = 50), patients with mild cognitive impairment (MCI, *n* = 50), and dementia (DEM, *n* = 50). The BOCA reliability was assessed using (i) Spearman’s correlation analysis between subscales; (ii) Cronbach’s alpha calculation, and (iii) Principal Component Analysis. Repeated-measures ANOVA was employed to assess the impact of the sequence of test administrations between the groups. BOCA performance between HS, MCI and DEM and within different severity subgroups were compared using Kruskall Wallis test. Furthermore, a comparison was conducted between MCI patients who tested positive for amyloid and those who tested negative, utilizing Mann Whitney’s U-test.

**Results:**

Test scores were significantly different between patients and controls (*p* < 0.001) suggesting good discriminative ability. The Cronbach’s alpha was 0.82 indicating a good internal consistency of the BOCA subscales and strong-to-moderate Spearman’s correlation coefficients between them. BOCA total and subscores differ across different MoCA severity subgroups and demonstrated strong correlation with MoCA scores (rho = 0.790, *p* < 0.001).

**Conclusions:**

The Italian version of the BOCA test exhibited validity, feasibility, and accurate discrimination closely performing as MoCA.

## Background

The assessment of cognitive decline and the prediction of dementia risk remain crucial aspects in addressing the challenges posed by an aging population. The expanding population of elderly individuals proficient in smartphone and digital technology offers significant opportunities for utilizing digital cognitive assessments in unsupervised settings (Öhman et al., 2021). Over the past decade, growing literature is showing the advantages of using digital devices for cognitive testing [1]. Integrating computerized cognitive tests in clinical practice may reduce time and costs associated with face-to-face testing and has several additional possible benefits. Indeed, digital cognitive assessment may provide standardized administration and automated scoring system, thus reducing inter-rater variability. The use of digital devices for cognitive testing in unsupervised conditions is also useful to reduce performance anxiety and the so-called “white-coat effect” observed in medical settings, and to provide a more accessible evaluation even in primary care settings. Computerized tests automatically record and store assessment data, thus reducing the likelihood of errors associated with manual data entry and facilitating easy access to longitudinal performance data for tracking changes over time [2–4]. The first step toward Digital Cognitive Assessment for adults and older adults during last year was the development of computerized administration and scoring of traditional paper-and-pencil screening tests, such as the Montreal Cognitive Assessment (MoCA). The MoCA is one of the most widespread and psychometrically robust screening tools for cognitive impairment, even in early stages. The MoCA is a rapid screening battery, which evaluates several cognitive domains (i.e., executive functioning, attention, language, memory, visuo-spatial abilities, orientation). In Italy, the MoCA has been adapted and standardized, and its clinical usability has been extensively examined [5–8]. Recently, a self-administered digital screening test called Boston Cognitive Assessment (BOCA) has been developed [9] demonstrating good internal consistency, adequate content validity, and strong test-retest reliability. BOCA demonstrated strong correlation with Montreal Cognitive Assessment (MoCA) and potentially represents a valid tool for early detection and monitoring of cognitive deficits, particularly in individuals at risk of cognitive impairments and dementia [9]. BOCA, being a digital screening tool, may enhance the efficiency of evaluations in clinical practice. First, the presence of an automatic scoring has many advantages, namely reduced costs and time associated with scoring procedure, reduced likelihood of errors, and a result easier to share with patients or caregivers. Second, the presence of non-repeating random stimuli prevents test-retest practice effect and is useful for monitoring patients’ cognitive performance over time. Third, BOCA allows the unsupervised administration, becoming a useful tool for screening in larger samples of patients and for further reducing the workload of the healthcare professionals.

In this study, we aim at evaluating the validity and feasibility of the Italian version of the BOCA in a large group of participants including healthy elderly, patients with mild cognitive impairment and patients with mild to moderate dementia.

## Methods

### Participants

The study included patients with cognitive deficits consecutively recruited by the Memory Clinic of the ASST Spedali Civili of Brescia and of the Neurology Unit of the University of Brescia and a group of controls matched for age, sex, and educational levels recruited among the patients’ caregivers. The study was approved by the Local Ethic Committee (NP 1471, DMA, Brescia, approved in its last version on April 19th, 2022). Each participant gave their informed consent prior to their inclusion. Cognitive impairment was defined by subjective cognitive complaints over a period of at least 6 months reported by the patient or caregivers. Among patients with cognitive deficits, individuals were assigned to the mild cognitive impairment (MCI) [10] group if their Clinical Dementia Rating scale (CDR) [11] was less than one; patients with CDR ≥ 1 were assigned into the Dementia group. Patients with MCI and dementia were further classified as amyloid-positive or -negative, according to the positivity of CSF pattern or amyloid-PET [12]. The functional abilities of participants were characterized using the basic activities of daily living (BADL) and the instrumental activities of daily living (IADL) [13].

Participants completed both BOCA and MoCA tests in a random order. The order-effect was assessed using a mixed effect model. All patients were unsupervised during the BOCA test administration, which was conducted in a quiet room at the hospital. Only a subset of cognitively healthy subjects completed the BOCA test at home. The BOCA test is automatically scored, and the total and subtest scores are provided immediately after the administration. Each participant’s report of the total and subtest scores was sent directly to the Memory Clinic by e-mail.

### BOCA test and items

BOCA is a 10-minute, self-administered test that includes randomly selected non-repeating items to minimize practice effects. BOCA includes eight subscales evaluating different cognitive domains, namely Memory/Immediate Recall, Memory Delayed/Recall, Executive Functions/Clock Test, Visuospatial Reasoning/Mental Rotation, Mental Math, Attention, Language/Prefrontal Synthesis, and Orientation. Better cognitive performance is indicated by higher scores, which have a maximum score of 30. Before each item, a set of announced instructions are provided verbally. The sound check at the beginning of the test ensures that users clearly hear the instructions. A detailed description of items is provided by Vyshedskiy et al. 2022 [9].

### Statistical analyses

The two-sample t-test and chi-squared test were used to assess differences in demographic variables for continuous and categorical variables, respectively. The Shapiro-Wilk test was used to assess normal distribution. All subtest scores did not meet the normality assumption with *p* < 0.05. To assess the correlation between BOCA and MoCA test scores, Spearman’s correlation was used with *p* < 0.05 as the statistical threshold. Linear regression was then calculated separately for patients and controls to assess the relationship between total BOCA score (independent variable) and participant age or education (dependent variables). BOCA reliability analysis was performed in several steps. First, the correlation matrix between the BOCA subscales was generated using Spearman’s correlation. Correlation strengths were determined as follows: 0.1–0.3 indicated a weak association, 0.31–0.50 a moderate association and 0.51-1.0 a strong association. Secondly, Cronbach’s alpha was obtained to determine the internal consistency of the BOCA test. Specifically, Cronbach’s alpha was derived considering all eight subscales. Then, one subscale at a time was removed and Cronbach’s alpha was recalculated for the remaining 7 subscales. All subscale scores were standardized for the purpose of this test. Thirdly, principal component analysis (PCA) was used to determine the number of components explaining the variance of the BOCA subscales. The Kaiser-Meyer-Olkin measure of sampling adequacy was used to test the assumptions. Mixed models were then used to simultaneously account for differences between diagnosis (between-subjects factor) and order of test administration (within-subjects). Differences in MoCA and BOCA scores between patients and controls, as well as within different equivalent scores classes were assessed using the Kruskal-Wallis non-parametric test. Post-hoc comparisons to assess differences between patients with MCI and dementia were performed using the Mann-Whitney U test. Moreover, the same statistical design was applied to assess differences between MCI patients who resulted amyloid positive vs. negative. All statistical analyses were performed using R version 4.3.1 and Jasp version 18.1. The statistical threshold was set at *p* < 0.05 for all tests.

### Data security

As previously described [9], the data in transit is encrypted using SSL. SSL stands for Secure Sockets Layer, a security protocol that creates an encrypted link between a web server and a web browser. SSL certificates secure online transactions and keep all information private and secure. The test data are stored in the secure cloud database in a reputable cloud provider.

## Results

### Baseline demographics and clinical features of the sample

BOCA and MoCA tests were administered to a total of 150 participants, namely 50 patients with MCI, 50 patients with dementia and 50 cognitively healthy controls, matched for age, sex, and educational levels (Table 1). Further, 33/50 (66%) of MCI and 49/50 (98%) of patients with dementia were classified as amyloid positive according to CSF amyloid-β 42 or amyloid-PET. MCI and Dementia patients differed from HC in all cognitive and functional measures (Table 1).

**Table 1:** Demographics and clinical characteristics in patients and controls

	Healthy Controls (*n* = 50)	MCI (*n* = 50)	Dementia (*n* = 50)	*p*-value
Age (mean yrs)	70.8 (11.9)	72.5 (6.9)	72.1 (9.6)	0.439
Education (mean yrs)	9.9 (4.1)	8.9 (3.4)	8.6 (3.2)	0.082
Female (%)	20 (38.5)	26 (52.0)	33 (61.1)	0.116
Clinical features				
CDR global	0.0 (0.0)	0.5 (0.0)	1.5 (0.5)	< 0.001
IADL (lost)	0.0 (0.0)	1.1 (0.2)	1.8 (0.4)	< 0.001
BADL (lost) BOCA Total Score MOCA Total Score	0.0 (0.0) 25.4 (3.5) 27.8 (1.6)	0.2 (0.4) 23.5 (2.2) 24.6 (3.4)	1.4 (0.6) 17.6 (2.9) 16.1 (3.3)	< 0.001 < 0.001 < 0.001
Amyloid positive (%)	-	33 (66)	49 (94)	< 0.001

Data are expressed as mean (SD). Abbreviations: CDR, Clinical Dementia Rating scale; IADL, Instrumental Activities of Daily Living; BADL, Basic Activities of Daily Living. IADL and BADL scores are expressed as skills lost as respect to the total. Amyloid-positivity was established based on CSF amyloid-β 42 < 650 or the presence of amyloid-plaques assessed with 11 C-PiB PET.

**Fig. 1:**
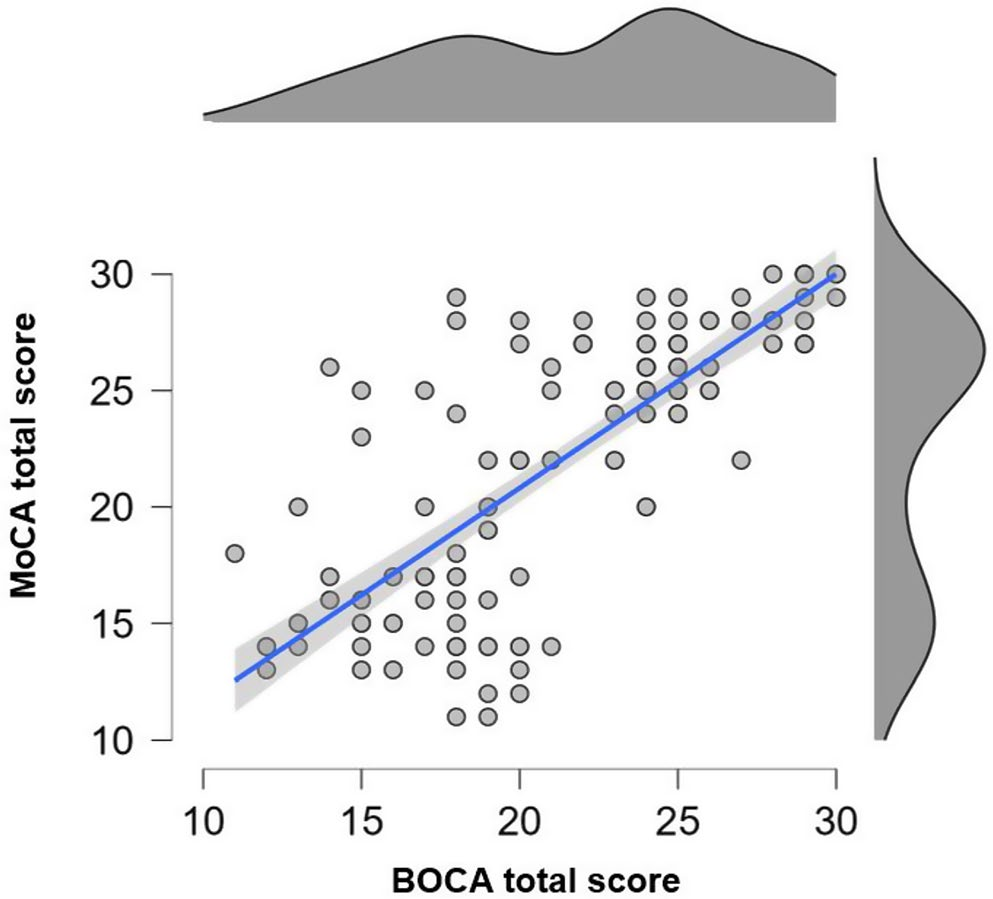
Correlation between MoCA and BOCA tests. Scatterplot representing the Spearman’s correlation between the two tests in the whole sample

Linear regression models revealed a significant relationship between BOCA total scores and age (β=-0.377, *p* = 0.005) or education (β = 0.322, *p* = 0.020) only in the control group. The Spearman’s Correlation revealed a positive and strong association between MoCA and BOCA total scores (rho = 0.790, *p* < 0.001, CI 95% [0.723, 0.837]) considering the whole cohort (Fig. 1).

### BOCA reliability analysis

**Fig. 2:**
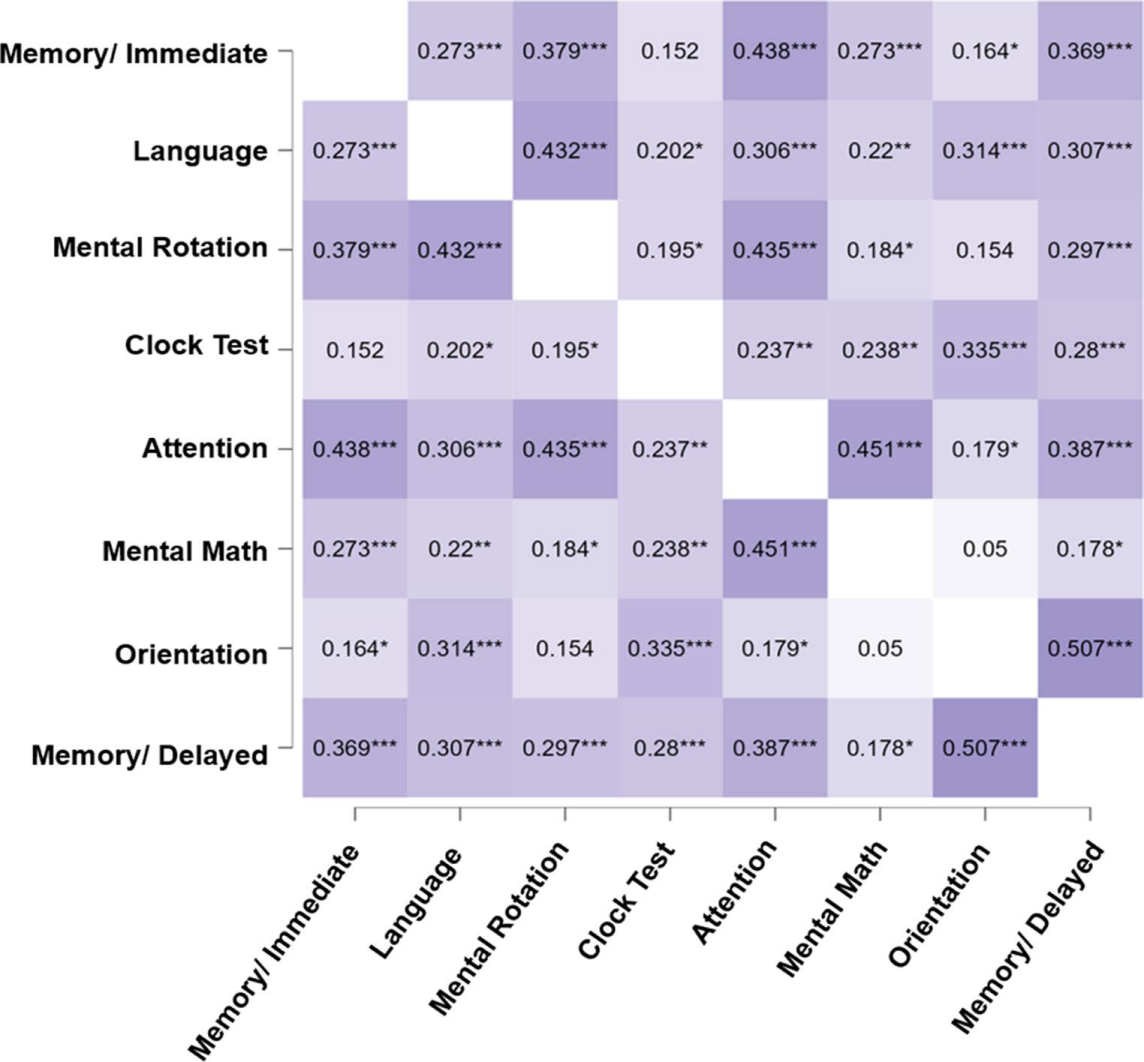
BOCA subscales correlation matrix. Heatmap representing significant Spearman correlations between BOCA subscales. Darker color represents a stronger relationship between variables. * = *p* < 0.05; **= *p* < 0.01; ***= *p* < 0.001

The correlation matrix for BOCA subscales was obtained via Spearman’s correlation method. The strongest correlation was between Orientation and Delayed Memory subscales (rho = 0.51, *p* < 0.001). Moderate associations were observed between Mental Math and Attention subscales (rho = 0.451, *p* < 0.001), Immediate Memory and Attention subscales (rho = 0.438, *p* < 0.001), Attention and Mental Rotation subscales (rho = 0.435, *p* < 0.001), and Mental Rotation and Language subscales (rho = 0.424, *p* < 0.001). Non-significant correlations were observed between Orientation and Mental Math subscales, and between Immediate Memory and Clock Test (see Fig. 2).

Internal consistency of the eight BOCA subscales was assessed using Cronbach’s alpha. Results indicated a good internal consistency (alpha = 0.822, CI 95% [0.775, 0.861]). Then, one subscale at time was removed, to re-calculate the Cronbach’s alpha for the remaining seven subscales. All subscales were standardized (i.e., z-scores) for this analysis. The resulting Cronbach’s alpha demonstrated high (> 0.796) internal consistency (Table 2).

**Table 2:** Internal consistency of the eight BOCA subscales

	Cronbach’s alpha (if item deleted)
Memory/ Immediate Recall	0.807
Language	0.810
Mental Rotation	0.806
Clock Test	0.817
Attention	0.796
Mental Math	0.819
Orientation	0.818
Memory/ Delayed Recall	0.807

The Kaiser-Meyer-Olkin measure of sampling adequacy indicated that the strength of the relationship between the subscales was high (KMO = 0.691) and the principal component analysis was possible. The eight subscales entered the model, and one component was retained using the eigenvalue > 1 criterion (eigenvalue = 2.89), explaining the 37% of variance. All subscales significantly correlated with the selected component (*p* < 0.05).

### Order effect assessment

Mixed models revealed no significant effect of the order of tests administration, also considering diagnosis as a between-subjects factor (BOCA total score: F = 0.042, *p* = 0.355; MoCA total score: F = 0.259, *p* = 0.772).

### Differences in the test scores between patients and controls

All participants completed MoCA and BOCA tests. The average MoCA score was 27.8 (SD = 1.6) in controls, 24.6 (SD = 3.4) in MCI and 16.1 (3.3) in patients with dementia. The MoCA total score was statistically different between the three considered groups (*p* < 0.001). Visuo-spatial, Calculation, Language Fluency, Abstraction, and Delayed Recall subscales were significantly lower in MCI patients as compared to controls. Each subscale significantly differentiated between patients with dementia and controls (Table 3).

**Table 3:** MoCA performance in patients and controls

MoCA Subscales	Healthy Controls (*n* = 50)	MCI (*n* = 50)	Dementia (*n* = 50)	*p*-value*	Post-hoc°
Visuospatial	4.8 (0.5)	4.4 (0.7)	3.1 (0.9)	< 0.001	HC > MCI HC > DEM MCI > DEM
Naming	2.9 (0.2)	2.9 (0.4)	2.3 (0.7)	< 0.001	HC > DEM MCI > DEM
Attention 1 and 2	2.8 (0.4)	2.7 (0.6)	1.8 (0.9)	< 0.001	HC > DEM MCI > DEM
Calculation	3.0 (0.2)	2.6 (0.6)	1.5 (0.7)	< 0.001	HC > MCI HC > DEM MCI > DEM
Language Repetition	1.5 (0.5)	1.5 (0.6)	1.0 (0.5)	< 0.001	HC > DEM MCI > DEM
Language Fluency	0.9 (0.3)	0.8 (0.4)	0.6 (0.5)	< 0.001	HC > MCI HC > DEM MCI > DEM
Abstraction	1.9 (0.3)	1.6 (0.5)	0.9 (0.4)	0.004	HC > MCI HC > DEM MCI > DEM
Delayed Recall	3.8 (1.1)	2.6 (1.4)	1.7 (1.0)	< 0.001	HC > MCI HC > DEM MCI > DEM
Orientation	5.9 (0.1)	5.6 (0.8)	3.3 (1.4)	< 0.001	HC > MCI HC > DEM MCI > DEM
MOCA Total Score	27.8 (1.6)	24.6 (3.4)	16.1 (3.3)	< 0.001	HC > MCI HC > DEM MCI > DEM

*= Kruskall-Wallis test, °= Mann-Whitney’s U test.

The average BOCA was 25.4 (SD = 3.5) for controls, 23.5 (SD = 2.2) for MCI patients, and 17.6 (SD = 2.9) for patients with dementia. The BOCA total score was significantly different between the three groups of individuals (*p* < 0.001). Immediate and Delayed Memory, Attention, and Mental Math subscales significantly distinguished MCI patients from controls. All BOCA subscale scores were also significantly different between patients with dementia and controls (Table 4).

**Table 4:** BOCA performance in patients and controls

BOCA Subscales	Healthy Controls (*n* = 50)	MCI (*n* = 50)	Dementia (*n* = 50)	*p*-value*	Post-hoc°
Memory/Immediate Recall	1.8 (0.6)	1.3 (0.8)	0.9 (0.7)	< 0.001	HC > MCI HC > DEM MCI > DEM
Language	4.6 (0.7)	4.2 (1.0)	3.7 (1.1)	< 0.001	HC > DEM MCI > DEM
Mental Rotation	2.4 (0.8)	2.1 (0.9)	1.4 (0.9)	< 0.001	HC > DEM MCI > DEM
Clock Test	2.9 (1.2)	2.9 (1.4)	1.5 (1.4)	0.001	HC > DEM MCI > DEM
Attention	2.9 (0.9)	2.4 (1.4)	1.9 (1.1)	< 0.001	HC > MCI HC > DEM MCI > DEM
Mental Math	3.6 (0.7)	3.3 (0.9)	2.8 (1.2)	0.002	HC > MCI HC > DEM MCI > DEM
Orientation	2.8 (0.4)	2.8 (0.5)	2.2 (0.8)	< 0.001	HC > DEM MCI > DEM
Memory/ Delayed Recall	4.4 (1.2)	4.1 (0.9)	3.2 (0.9)	< 0.001	HC > MCI HC > DEM MCI > DEM
BOCA Total Score	25.4 (3.5)	23.5 (2.2)	17.6 (2.9)	< 0.001	HC > MCI HC > DEM MCI > DEM

*= Kruskall-Wallis test, °= Mann-Whitney’s U test.

Independently from final diagnosis (HC, MCI or dementia), BOCA total and subitems scores distribution was contrasted in subjects with different equivalent scores based on North Italy normative data (Aiello et al. 2022). The results, highlighted in Table 5, showed differences in BOCA total scores among all subgroups, all subitems scores were different between Equivalent score (ES) 4 from 0, whereas most of subitems also differentiated different ES subgroups (see Table 5 for details).

**Table 5:** Comparison of BOCA total score and subscale according to MoCA equivalent scores based on Italian validation of Aiello and Coauthors [7]

	ES = 0 (*n* = 40)	ES = 1 (*n* = 10)	ES = 2 (*n* = 10)	ES = 3 (*n* = 8)	ES = 4 (*n* = 86)	*p*-value*
BOCA total score	16.41 (2.5)	17.70 (3.6)	20.30 (4.7)	21.13 (2.6)	25.04 (3.41)	< 0.001^a, b,c^
Memory/Immediate Recall	0.76 (0.7)	1.30 (0.8)	1.30 (0.8)	1.00 (0.7)	1.54 (0.7)	< 0.001^d^
Language	3.59 (1.1)	3.10 (1.4)	3.90 (1.1)	4.38 (0.8)	4.48 (0.8)	< 0.001 ^d, e^
Mental Rotation	1.24 (0.8)	1.60 (0.9)	2.10 (0.9)	1.13 (1.3)	2.39 (0.8)	< 0.001 ^c, d^
Clock Test	1.29 (1.4)	1.40 (1.3)	2.10 (1.7)	2.65 (1.5)	3.05 (1.2)	< 0.001 ^d, e^
Attention	1.78 (1.1)	1.90 (1.2)	1.60 (1.4)	1.63 (1.1)	2.85 (1.1)	< 0.001 ^b, c,d^
Mental Math	2.67 (1.2)	2.80 (1.1)	2.50 (1.2)	3.25 (0.9)	3.63 (0.6)	< 0.001 ^c, d^
Orientation	2.03 (0.8)	2.00 (0.8)	2.70 (0.5)	3.00 (0.0)	2.85 (0.4)	< 0.001 ^c, d^
Memory/ Delayed Recall	3.03 (0.9)	3.60 (0.7)	4.10 (0.9)	4.13 (0.6)	4.26 (1.1)	< 0.001 ^a^

Abbreviations: ES, equivalent score. ^a^=PE 0 ≠ PE 2,3,4; ^b^= PE 2 ≠ PE 4; ^c^=PE 3 ≠ PE 4; ^d^=PE 0 ≠ PE 4; ^e^=PE 1 ≠ PE 4.

### Differences between amyloid positive and negative MCI patients

MCI amyloid-positive (MCI-Amy+, *n* = 33) and MCI amyloid-negative (MCI-Amy-, *n* = 17) were compared as for BOCA and MoCA. These two subgroups were comparable for age, sex, and education level. As for the MoCA test, only Abstraction subscale was significantly more impaired in MCI-Amy + than MCI-Amy- (*p* = 0.014). The MoCA total score in MCI-Amy + was not statistically different from in MCI-Amy- (*p* = 0.315).

Memory Immediate Recall (*p* = 0.004), Memory Delayed recall (*p* = 0.023), and Mental Rotation (*p* = 0.045) BOCA subscales were significantly more impaired in MCI-Amy + than MCI-Amy-. Consistently, the BOCA total score was lower in MCI-Amy + than MCI-Amy- (*p* = 0.048).

## Discussion

The study focused on the validation and feasibility of the Boston Cognitive Assessment (BOCA) in the Italian population, in a large set of participants with normal cognition, MCI and mild to moderate dementia. The results of the study indicated a strong correlation between BOCA and MoCA total scores, showcasing good concurrent validity. The internal consistency of BOCA subscales was found to be high, with significant correlations observed between them. Both MoCA and BOCA tests were effective in differentiating between groups of healthy individuals, mild cognitive impairment, and dementia. Further, the performance of BOCA was also high according to MOCA stratification by using Italian normative data [9, 14]. These data agree with previous validation studies [9, 14] and support the usefulness of BOCA as a tool for the detection of cognitive impairment.

Over the last decade, there has been a remarkable increase in the development of digitalized tools for cognitive assessment [4, 15–17]. Of note, there is a growing interest in the development and integration of tablet-based cognitive tools in clinical practice, particularly among adults and elderlies. Indeed, prior evidence suggests that adults over age 55 have a great preference for use of tablet devices [18]. The administration of tablet-based cognitive tests is standardized, minimizing the subjective influences of examiners and ensuring consistent evaluation across participants. Still, the application of such digital testing is limited in clinical practice due to lack of feasibility studies testing their construct validity, applicability, and discriminative performances in real-world populations [19]. In this study, 150 participants (50 healthy controls, 50 participants with MCI, and 50 with dementia) were included and underwent MoCA and unsupervised BOCA testing in a randomized order. The general applicability of the test was high, with no patients refusing the test or not being able to complete it by themself. BOCA and MoCA total scores demonstrated high correlation when considering the entire cohort. The internal consistency of BOCA subscales was high as demonstrated by the overall Cronbach’s alpha, even when each subscale was removed one at a time. Moreover, the principal component analyses revealed that BOCA subscales collectively represent a single underlying factor that explains a significant portion of the variability in the data. The results are in agreement with the original validation performed by Vyshedskiy and coauthors (2022) [9] in the English version of BOCA and showed high applicability even in patients with MCI and dementia. In additional analyses based on normative Italian data [9, 14], BOCA was able to differentiate subgroups of individuals with different equivalent scores (i.e. severity degrees) arguing for a comparable efficacy in detecting cognitive abnormalities. Relative to the control group, a significant relationship between BOCA total scores and both age and education was found, as revealed by linear regression models. This finding is partially in contrast with previous literature in which BOCA was not found to be significantly associated with demographic characteristics [14], whereas more recent validation studies showed an effect of education in the English version of BOCA [20].

Additionally, three BOCA subscales (Memory Immediate Recall, Memory Delayed recall, and Mental Rotation) were notably lower in amyloid-positive patients compared to amyloid-negative individuals. While this alone may not be sufficient to determine BOCA’s ability to identify MCI due to Alzheimer’s Disorder (AD), it sheds light on the potential of the Boston Cognitive Assessment (BOCA) to discern subtle cognitive differences potentially associated with AD to be verified in longitudinal studies [21].

Several limitations of the study should be acknowledged. Firstly, the sample size was fair but still limited, which may restrict the generalizability of our findings. Although we matched participants based on age, sex, and education level to mitigate potential confounding variables, a larger sample size would enhance the robustness of our results and allow for more comprehensive subgroup analyses. Future studies with a larger sample size are still an urgent need in order to provide Italian normative data and cut-off to distinguish patients from healthy ageing individuals. For this purpose, the recruitment of patients and healthy-ageing individuals is still ongoing. Another limitation is the lack of a control group with completely remote home-testing. It is important to consider the potential for uncontrolled bias in home-based settings, which may differ from the application in a quiet hospital room. Future research should compare BOCA scores obtained in controlled clinical settings with those obtained in at-home environments to assess potential discrepancies and identify strategies to minimize distractions during self-administration.

Additionally, it should be noted that our study primarily focused on patients with mild cognitive impairment (MCI) and dementia. Therefore, the generalizability of our findings to other patient populations may be limited. To address this limitation, future research should investigate BOCA scores in diverse patient groups, such as those with stroke, traumatic brain injury, aphasia, and other neurological disorders. Comparing BOCA scores across different patient populations would provide valuable insights into the test’s sensitivity and specificity across a range of cognitive performances.

Finally, it may be worth considering conducting test-retest reliability analyses in future studies, although the literature suggests good long-term stability and the absence of a learning effect [9]. The ongoing longitudinal study of controls, MCI, and dementia will help determine long-term stability and the potential of BOCA as a tool for tracking cognitive changes over time.

## Conclusions

The study suggests that the Italian version of BOCA is a valid and applicable tool with a high discriminative ability for assessing cognitive status across the spectrum from normal cognition to dementia. The findings highlight the significance of early detection and monitoring of cognitive decline and suggest that BOCA could be a feasible and effective screening and monitoring tool in the population due to its stability and cost-efficiency. However, it is important to note that the study suggests that further extensive validation is necessary to explore the cost-effectiveness and applicability of BOCA as a cognitive assessment tool in aging individuals. This indicates the potential impact it may have on enhancing cognitive health assessments and care for aging populations.

## References

[1] Koo BM, Vizer LM (2019) Mobile technology for cognitive assessment of older adults: a scoping review. Innov Aging 3:igy038 30619948 10.1093/geroni/igy038 PMC6312550

[2] Samaroo A, Amariglio RE, Burnham S Diminished learning over repeated exposures (LORE) in preclinical Alzheimer’s disease. Alzheimer’sDementia:, Diagnosis et al (2020) Assessment & Disease Monitoring 12:e12132 10.1002/dad2.12132 PMC7784542 33426266

[3] Öhman F, Hassenstab J, Berron D Current advances in digital cognitive assessment for preclinical Alzheimer’s disease. Alzheimer’sDementia:, Diagnosis et al (2021) Assessment & Disease Monitoring 13:e12217 10.1002/dad2.12217 PMC8290833 34295959

[4] Zygouris S, Tsolaki M (2015) Computerized cognitive testing for older adults: a review. Am J Alzheimers Dis Other Demen 30:13–28 24526761 10.1177/1533317514522852 PMC10852880

[5] Santangelo G, Siciliano M, Pedone R et al (2015) Normative data for the Montreal Cognitive Assessment in an Italian population sample. Neurol Sci 36:585–591 25380622 10.1007/s10072-014-1995-y

[6] Conti S, Bonazzi S, Laiacona M et al (2015) Montreal Cognitive Assessment (MoCA)-Italian version: regression based norms and equivalent scores. Neurol Sci 36:209–214 25139107 10.1007/s10072-014-1921-3

[7] Aiello EN, Pasotti F, Appollonio I, Bolognini N (2022) Equating mini-mental state examination (MMSE) and Montreal cognitive assessment (MoCA) scores: conversion norms from a healthy Italian population sample. Aging Clin Exp Res 34:1721–1724 35182351 10.1007/s40520-022-02089-w

[8] Bosco A, Spano G, Caffò AO et al (2017) Italians do it worse. Montreal Cognitive Assessment (MoCA) optimal cut-off scores for people with probable Alzheimer’s disease and with probable cognitive impairment. Aging Clin Exp Res 29:1113–1120 28155182 10.1007/s40520-017-0727-6

[9] Vyshedskiy A, Netson R, Fridberg E et al (2022) Boston cognitive assessment (BOCA)—a comprehensive self-administered smartphone-and computer-based at-home test for longitudinal tracking of cognitive performance. BMC Neurol 22:92 35291958 10.1186/s12883-022-02620-6 PMC8922721

[10] Petersen RC, Negash S (2008) Mild cognitive impairment: an overview. CNS Spectr 13:45–53 18204414 10.1017/s1092852900016151

[11] Hughes CP, Berg L, Danziger W et al (1982) A new clinical scale for the staging of dementia. Br J Psychiatry 140:566–572 7104545 10.1192/bjp.140.6.566

[12] Padovani A, Caratozzolo S, Rozzini L et al (2022) Real-world eligibility for aducanumab depends on clinical setting and patients’ journey. J Am Geriatr Soc 70:626 34750815 10.1111/jgs.17530 PMC9298825

[13] Lawton MP, Brody EM (1969) Assessment of Older people: self-maintaining and instrumental activities of daily Living1. Gerontologist 9:179–186. 10.1093/geront/9.3_Part_1.179 5349366

[14] Gold D, Stockwood J, Boulos K et al (2022) The Boston cognitive assessment: psychometric foundations of a self-administered measure of global cognition. Clin Neuropsychol 36:2313–2330 34075854 10.1080/13854046.2021.1933190

[15] Padovani A, Pilotto A (2023) Digital Technologies in Cognitive Disorders. In: Gerontechnology. A Clinical Perspective. Springer, pp 87–97

[16] Perin S, Buckley RF, Pase MP et al (2020) Unsupervised assessment of cognition in the Healthy Brain Project: implications for web-based registries of individuals at risk for Alzheimer’s disease. Alzheimer’s & Dementia: Translational Research & Clinical Interventions 6:e12043 10.1002/trc2.12043 PMC7317647 32607409

[17] Kourtis LC, Regele OB, Wright JM, Jones GB (2019) Digital biomarkers for Alzheimer’s disease: the mobile/wearable devices opportunity. NPJ Digit Med 2:9 31119198 10.1038/s41746-019-0084-2 PMC6526279

[18] Canini M, Battista P, Della Rosa PA et al (2014) Computerized neuropsychological assessment in aging: testing efficacy and clinical ecology of different interfaces. Comput Math Methods Med 2014:804723 25147578 10.1155/2014/804723 PMC4131509

[19] Cubillos C, Rienzo A (2023) Digital cognitive assessment tests for older adults: systematic literature review. JMIR Ment Health 10:e47487 38064247 10.2196/47487 PMC10746978

[20] Ferguson H, Turok N, Gold D et al (2022) A-303 preliminary analysis of the influence of Age and Education on the Boston Cognitive Assessment (BoCA). Arch Clin Neuropsychol 37:1457

[21] Williams OA, An Y, Armstrong NM et al (2020) Profiles of cognitive change in preclinical and prodromal Alzheimer’s disease using change-point analysis. J Alzheimer’s Disease 75:1169–1180 32390623 10.3233/JAD-191268 PMC7561016

